# Association of Gestational Diabetes Mellitus (GDM) with subclinical atherosclerosis: a systemic review and meta-analysis

**DOI:** 10.1186/1471-2261-14-132

**Published:** 2014-09-29

**Authors:** Jing-Wei Li, Si-Yi He, Peng Liu, Lin Luo, Liang Zhao, Ying-Bin Xiao

**Affiliations:** Institute of Cardiovascular Surgery, PLA, Xinqiao Hospital, Third Military Medical University, No. 183 Xinqiao Street, Chongqing, 400037 PR China

**Keywords:** Gestational diabetes mellitus, Carotid intima-media thickness, Atherosclerosis

## Abstract

**Background:**

Gestational diabetes mellitus (GDM) is associated with an elevated risk of adverse health outcomes such as type 2 diabetes and cardiovascular diseases. Carotid intima-media thickness (cIMT) is increasingly used as a noninvasive marker for subclinical atherosclerosis. Whether there is a direct correlation between GDM and elevated cIMT is still controversial.

**Methods:**

PubMed, Embase and reference lists of relevant papers were reviewed. Studies assessing the relationship between GDM and cIMT were included. Weighted Mean Difference (WMD) of cIMT was calculated using random-effect models.

**Results:**

Fifteen studies with a total of 2247 subjects were included in our analysis, giving a pooled WMD of 0.05 (95% confidence interval [CI] 0.03 –0.07). Furthermore, meta regression and subgroup analysis found that the association between GDM and larger cIMT already existed during pregnancy, and this relation was stronger in obese GDM patients.

**Conclusions:**

GDM in and after pregnancy is associated with subclinical atherosclerosis. Weight control may be helpful to prevent cardiovascular diseases for GDM patients.

**Electronic supplementary material:**

The online version of this article (doi:10.1186/1471-2261-14-132) contains supplementary material, which is available to authorized users.

## Background

Gestational diabetes mellitus (GDM) is one of the common complications during pregnancy, which incidence is approximately 5% (range from 1 to 14%) and this number is increasing due to increased prevalence of obesity [1]. GDM women have an increased risk for type 2 diabetes mellitus, cardiovascular disease and metabolic syndrome years after pregnancy, also offspring of GDM women have a higher risk for noncommunicable diseases and obesity rates [2].

Carotid intima-media thickness (cIMT) is measurement of the combined thickness of the intimal and medial layers of the carotid artery by B-mode ultrasound. cIMT is a noninvasive technique to dectect subclinical atherosclerosis [3], and is associated with multiple cardiovascular risk factors [4], cardiovascular events [5] and coronary artery diseases [6].

As GDM alone is independent predictors of obstructive coronary artery disease [7] and cardiovascular diseases. We suspect whether there is a direct correlation between GDM and elevated cIMT. However, studies focusing on this issue have been small and have reported conflicting results. Therefore, we conducted a meta-analysis to assess the correlation between GDM and cIMT.

## Methods

### Literature search

We searched the databases of EMBASE and PubMed and references lists of relevant papers to MAY 24, 2014. EMBASE search terms were ‘pregnancy diabetes mellitus’/exp and ‘arterial wall thickness’/exp. Similar search terms were used for PubMed. The search strategy (Additional file 1) has been put into the supplemental material. No language and time limitation was performed.

### Study selection

We selected published trials that investigated the relationship between gestational diabetes and cIMT. Excluded were (1) studies published as conference articles; (2) cIMT was not measured in both gestational diabetes and control groups; and (3) reports having duplicate study population. All literature searches were independently reviewed by 2 authors (JW L and SY H) to identify relevant trials that met the inclusion criteria. Disparities were adjudicated by a third author (YB X). For each included article, study characteristics, including authors, publication year, country, ages, duration, BMI, mean and standard deviation of CIMT were extracted independently by two researchers (JW L and SY H). If the studies were studying the same population, we included the newer and completed ones in this meta-analysis.

### Statistical analysis

The cIMT in both gestational diabetes and control groups were induced to our meta-analysis. Statistical heterogeneity between studies was tested by Cochran’s test (P < 0.05). We used the random-effect model in this meta-analysis, which takes into account heterogeneity among studies, because the study design and measuring time were different across studies. The Cochrane Q test and I^2^ was used to evaluate the presence of heterogeneity. If heterogeneity exists, subgroup analyses were conducted to evaluate effect modification by study-level characteristics including publish year, number of patients, ages at pregnancy, measuring time (in pregnancy or after pregnancy), BMI and duration. Publication bias was assessed with Egger’s test. All statistical significance was set at a p value of 0.05, and CIs were calculated at the 95% level. Statistical analyses were performed with Stata software (version 11.0; Stata Corporation, College Station, TX).

## Results

### Search results and study characteristics

**Figure 1:**
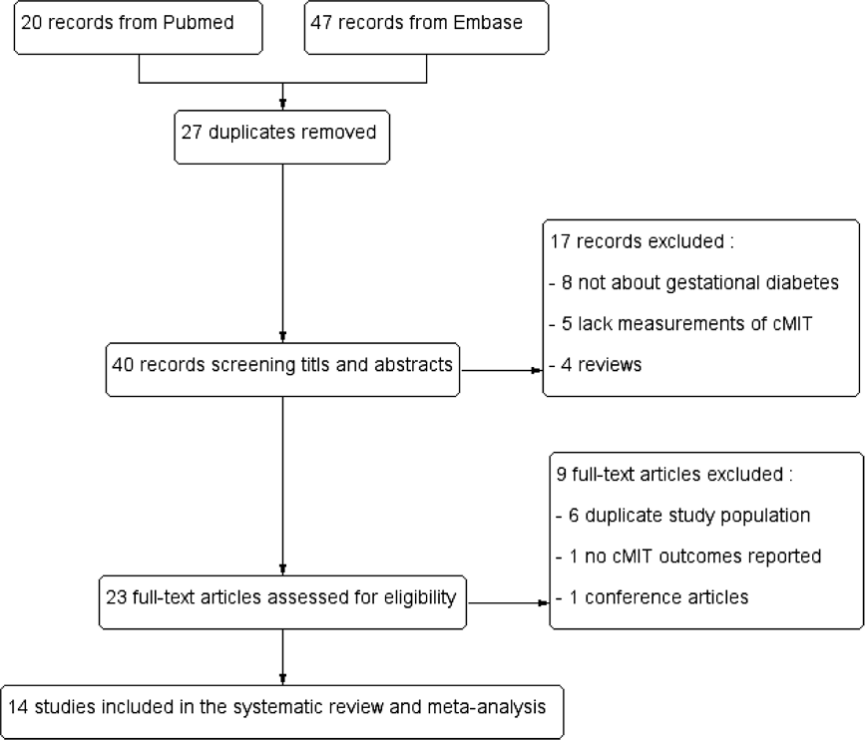
**Literature search and selection process.**

A total of 67 articles were identified in a combined search of PubMed and EMBASE. We also manually searched studies cited in previous reviews and of references list from retrieved articles. First 27 duplicates were removed, and then 18 articles were initially excluded through screening title and abstract. Among the 23 articles retrieved for further review of the full text, 6 were excluded for repeated reports, 1 for not reporting cIMT outcomes, and 1 study for conference reports. Akinci B and his colleagues investigated the association between GDM and CVD from different aspects and published five articles using the same population [1, 8–11]. Mehmet Vural and his colleagues [12] studied the same population with Mehmet Ali Eren [13]. Eventually, 15 studies with a total of 2247 subjects were included in our meta-analysis (Figure 1) [11, 13–26]. Study characteristics and exclusion criteria included in the analysis are shown in Table 1. Only the study of Gunderson [19] was evalauted at multivariate analysis (adjusted for age, race, parity, pre-pregnancy BMI, HOMA-IR, weight gain, year 20-HOMA-IR + DBP, incident diabetes and metabolic syndrome), other studies used unadjusted data. Other characteristics of included studies have been put into the supplemental material (Additional file 2).

**Table 1:** **Study characteristics of included studies**

Author	Age	No. patient	Country	Duration(year)	BMI		Waist		Exclusion:
					GDM	CG	GDM	CG	
Baris Akinci [11]	35.1	190	Turkey	3.39	26.82 ± 4.25	26.5 ± 2.66	90.31 ± 11.68	87.45 ± 8.93	known cardiovascular disorders, type 1 or type 2 diabetes (diagnosed before the index pregnancy), familial hyperlipidemia, hypertension, acute infection, chronic inflammatory disease, coagulation disorders and other systemic diseases, on peri- or postmenopausal period at the time of sampling
A.E. Atay [14]	27.9	75	Turkey	2.29	32.2 ± 4.8	27.3 ± 4.2			receiving any medication during the last 3 months, with liver or renal dysfunction, hyperprolactinemia, or thyroid disease and smokers, with GDM and healthy pregnant women with a history of GDM in their previous pregnancies or glucose tolerance before the present pregnancy, healthy pregnant women with a family history of DM
S. Bo [15]	41.1	195	Italy	6.5	Group 1: 20.2 ± 2.2 Group 2: 23.6 ± 5.2	22.1 ± 3.1	Group 1: 73.6 ± 5.7 Group 2: 86.4 ± 13.5	79.9 ± 9.9	known pre-pregnancy conditions, such as diabetes mellitus, diseases affecting glucose metabolism, hypertension, chronic illness, and medical treatments (including hormonal preparations), presence of a positive OGCT, but an OGTT not diagnostic for GDM.
Mustafa Caliskan [16]	33.4	95	Turkey	6	26.9 ± 3.9	26.1 ± 2.7	85.0 ± 5.9	84.4 ± 4.9	presence of a valvular or congenital heart disease; cardiac rhythm other than sinus; previous myocardial infarction; hypo- or hyperthyroidism; chronic obstructive pulmonary disease or corulmonale; systemic diseases (etc. hemolologic ,hepatic, and renal diseases) or any disease that could impair coronaryflow reserve; hypertrophic cardiomyopathy; family history of coronary artery disease; excessive alcohol consumption (>120 g/day); previous lipid metabolism disorders; history of dyslipidemia; smoking; and diabetes mellitus.those with ST segment or T-wave changes specific for myocardial ischemia, Q-waves, and incidental left bundle branch block on ECG
Mehmet Ali Eren [13]	31	64	Turkey	0	31.8 ± 5.5	29.4 ± 5.4			smoking, alcohol abuse, preeclampsia, multiple pregnancies, pregestational diabetes for all study participants, and a family history of diabetes mellitus (for the control group only), pregnancies with GDM who had overt diabetes with 75-g standard OGTT in the 6-week after delivery
Hossein Fakhrzadeh [17]	33	40	Iran	4	27.63 ± 3.52	27.33 ± 5.64			current or previous smokers, patients who had pre-existing HTN, diabetes mellitus (DM), and women with symptomatic CVD
Claudia Maria Vilas Freire [18]	35.7	139	Brazil	2.7	29.01 ± 0.66	22.46 ± 0.42	92.09 ± 1.63	74.08 ± 1.14	any past condition afflicting them at previous pregnancies, other than GDM, was considered an exclusion criteria, especially those requiring hospital admission such as preeclampsia. alcoholism, drug addiction, uremia as well as those with liver, psychiatric, rheumatologic, and thyroid diseases or in use of corticosteroids
Erica P. Gunderson [19]	44.2	898	USA	20	24.8 (5.6)	23.3 (4.3)	74.4 (11.1)	71.7 (8.8)	heart disease or diabetes before pregnancies andthose without any post-baseline births,missing ccIMT measurements, and with history of heart disease,recently or currently pregnant, and with previous hysterectomy at baseline, with clinically relevant diabetes at baseline and those who developed diabetes before the first post-baseline birth
H Ijas [20]	52.2	116	Finland	19	27.1 ± 5.3	24.5 ± 4.2	94.4 ± 14.9	94.4 ± 14.2	GDM diagnosed in their subsequent pregnancy
Ufuk Ozuguz [22]	30.1	101	Turkey	0	29.95 ± 4.21	26.34 ± 4.08			previously knowndiagnosis of diabetes mellitus; the presence of an additional cardiovascular risk factor such as hypertension, hyperlipidemia or coronary artery disease; presence of other factors that may affect serum lipid profile and/or hsCRP level (acetylsalicylic acid, smoking, impaired liver and kidney functions, history of trauma, an acute infection within one month prior to presentation or a chronic infection); presence of an underlying chronic inflammatory condition such as collagen tissue and inflammatory bowel diseases.
E. TARIM [23]	29.4	70	Turkey	0	28.65 ± 4.75	27.17 ± 2.90			smokers, patients who had folic acid and vitamin B12 deficiency, hypertension, multiple pregnancy, fetal abnormalities, pre-existing hypertension and diabetes, thyroid disease or a history of significant severe diseases, family history of coronary heart disease and stroke
I Vastagh [24]	32.2	42	Hungary	0	28 ± 4	27 ± 4			have a history of diabetes mellitus or a previous GDM.
Gholamreza Yousefzadeh [26]	24.8	50	Iran	0	28.7 ± 4.5	26.5 ± 4.5			family history of cardiovascular disorders; history of hypertension; anti-hypertensive and cholesterol medication use; hyperlipidemia; overt diabetes or fasting plasma glucose (FPG) > 125 mg/dl;chronic renal or hepatic diseases; malignancies; recent hormonal medications; cigarette smoking; severe obesity (body mass index [BMI] >35 kg/m2); and history of infertility or polycystic ovarian disease, with the status of plaques/shadowing ( > 1.0 mm) at any carotid site
Volpe, L. [25]	36.3	52	Italy	2	25.7 ± 8.9	23 ± 3.4	86.9 ± 9.7	79.6 ± 9.7	not mentioned
Yun Hyi Ku [21]	32.3	120	Korea	1	22.3 (20.4-24.2)	20.4 (19.5-23.1)	80.3 ± 7.7	74.5 ± 7.7	females who were diagnosed with gestational diabetes between the 24th and 28th week of pregnancy

### GDM is associated with cIMT

**Figure 2:**
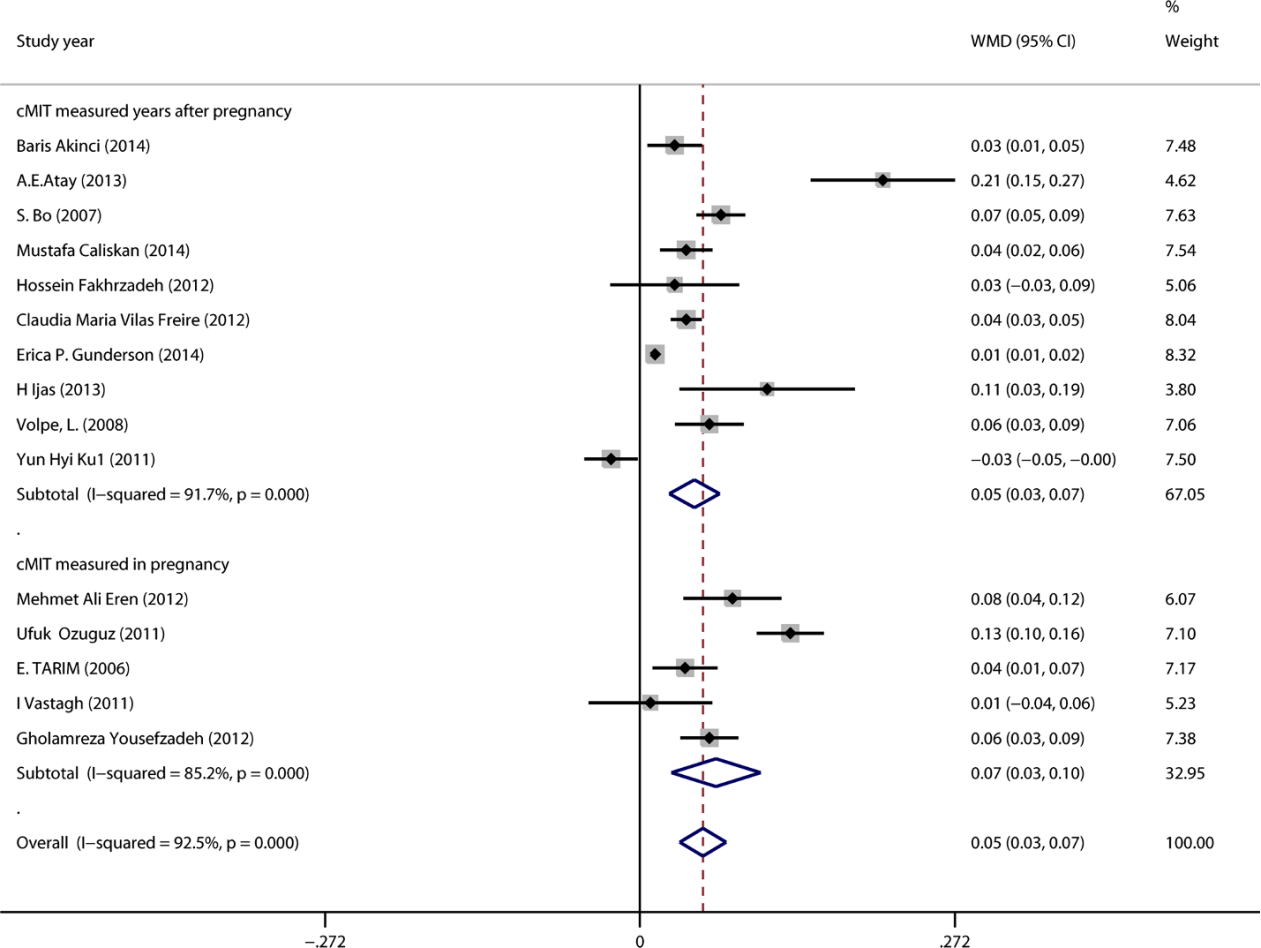
**Forest plots showing effects of GDM on cIMT.**

**Figure 3:**
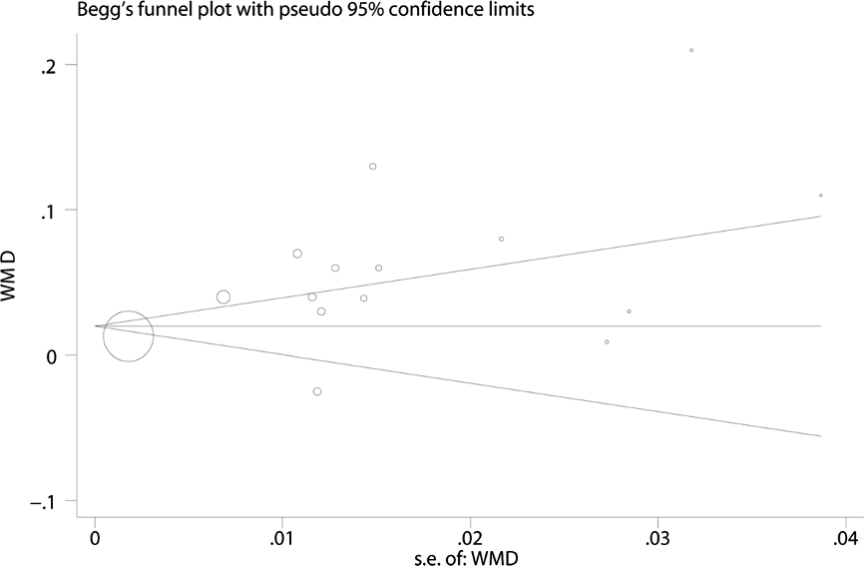
**Begg’s funnel plot showing publication bias.**

The cIMT from both GDM and control groups was pooled. The WMD was 0.05 (95% CI: 0.03–0.07, P < 0.001). The statistic value I^2^ was 92.5%, P < 0.001 (Figure 2). No significant publication bias was found for WMD by Begg’s test (P = 0.621) (Figure 3). We performed meta-regression analyses on cIMT to investigate the cause of heterogeneity, and found the BMI may be one of the main causes (P = 0.048, Table 2). Subgroup analysis was performed to distinguish the heterogeneity among these studies. Results showed that study object with higher BMI got larger cIMT (WMD: 0.07, 95% CI: 0.03–0.12 for those with BMI > 27.6 and WMD: 0.04, 95% CI: 0.02–0.06 for those with BMI < 27.6). Diagnostic criteria of GDM might influence the results (WMD: 0.08, 95% CI: 0.05–0.11 for Carpenter and Coustan criteria, WMD: 0.03, 95% CI: −0.01–0.07 for NDDG criteria, WMD: 0.04, 95% CI: −0.01–0.09 for WHO criteria and WMD: 0.01, 95% CI: −0.06–0.07 for ADA 75 g criteria). There seemed no difference as to measuring time of cIMT with GDM (WMD: 0.07, 95% CI: 0.03–0.10 when measured in pregnancy and WMD: 0.05, 95% CI: 0.03–0.07 when measured years after pregnancy) and ages at pregnancy (WMD: 0.07, 95% CI: 0.03–0.11 for those with age < 31 and WMD: 0.04, 95% CI: 0.02–0.07 for those with age > =31). The GDM did not significantly increase cIMT as to publish year (WMD: 0.07, 95% CI: 0.03–0.10 for those published after 2013 and WMD: 0.05, 95% CI: 0.02–0.08 for those before 2013), number of patients (WMD: 0.06, 95% CI: 0.03–0.08 for number of patients above 90 and WMD: 0.05, 95% CI: 0.02–0.07 for number of patients below 90) and duration between the time of GDM diagnosed and cIMT measured (WMD: 0.05, 95% CI: 0.01–0.09 for duration > 4 and WMD: 0.05, 95% CI: 0.01–0.09 for duration between 0 and 4) (Table 3).

**Table 2:** **Results of meta regression of GDM on cIMT**

Item	Coef	P	95% CI	
Publish year	.0005284	0.930	-.0121875	.0132443
Age at pregnancy	-.0017171	0.704	-.0112528	.0078185
No. Patients	-.0000597	0.372	-.0001991	.0000797
BMI of GDM	.0100715	0.048	.0001263	.0200168
Measuring time	.013057	0.676	-.0529943	.0791082
Duration	-.0005414	0.823	-.0056708	.0045881
GDM Criteria	-.0234964	0.062	-.0484176	.0014249

Coef = regression coefficients.

CIs = confidence intervals.

**Table 3:** **Stratified analyses of GDM on cIMT**

Item	Subgroup	No	WMD	95% CI		P. Het	I ^2^	P. test
Publish year	> = 2013	5	0.07	0.03	0.10	<0.001	92.2	0.001
	<2013	10	0.05	0.02	0.08	<0.001	88.8	<0.001
No. pat	> = 90	8	0.05	0.02	0.07	<0.001	94.3	<0.001
	<90	7	0.07	0.03	0.10	<0.001	79.9	<0.001
Ages at pregnancy	> = 31	8	0.04	0.02	0.07	<0.001	85.4	0.001
	<31	7	0.07	0.03	0.11	<0.001	94.9	<0.001
Measuring time	In pregnancy	5	0.07	0.03	0.10	<0.001	91.7	<0.001
	After pregnancy	10	0.05	0.03	0.07	<0.001	85.2	0.001
Duration (years)	>4	4	0.05	0.01	0.09	<0.001	92.1	0.007
	>0, <4	6	0.05	0.01	0.09	<0.001	91.6	0.010
BMI	> = 27.6	7	0.07	0.03	0.12	<0.001	95.1	0.001
	<27.6	8	0.04	0.02	0.06	<0.001	83.8	0.001
GDM Criteria	Carpenter	7	0.08	0.05	0.11	<0.001	90.5	<0.001
	NDDG	3	0.03	<−0.01	0.07	0.001	85.2	0.072
	WHO	2	0.04	−0.01	0.09	0.102	62.6	0.114
	ADA-75 g	4	0.01	−0.06	0.07	<0.001	93.5	0.816

## Discussion

During pregnancy, insulin resistance increases. In healthy women compensatory insulin secretion counteracts this demand, while in GDM patients, not enough insulin is secreted to overcome the insulin demand. Compared with healthy ones, GDM patients are more likely to have type 2 diabetes and endothelial dysfunction, known conditions that leads to higher risk for cardiovascular diseases [27].

The results from our systematic review and meta-analyses indicate GDM was significantly associated with elevated cIMT, and this association already exists at the time of pregnancy. Fatty women with GDM seem to have larger cIMT.

Most of our included studies have found GDM is associated with larger cIMT. The study by A.E. Atay et al. [14] was the one finding the most significant difference of cIMT between GDM patients and control. The study population included in this study was fatter (BMI: 32.2 ± 4.8 for the GDM group vs 27.3 ± 4.2 for the control). Earlier study has found that obese patients with GDM had higher prevalence of chronic hypertension [28] and cardiovascular disease [29]. Our meta-regression and subgroup analyses confirms that the association between GDM and cIMT is influenced by BMI. The study of H Ijas et al. [20] showed that GDM patients with BMI > 25 had larger cIMT compared with those with BMI < 25 and controls. Also Gunderson and his colleagues [19] has found the association between GDM and cIMT changed from significance to insignificance after adjusting BMI. The study by Yun Hyi Ku [21] found there was no association between GDM and cIMT. As this study was conducted in Korea, the author compared their results with western ones and inferred it may be caused by culturally-based obesity. The author explained that as obesity was one of the major factor influencing cIMT, obesity is much less common in their country than in western ones ( BMI of study objects were in normal range), which may lead to insignificance of their results. Contrary to these findings, the study of S. Bo et al. [15] found that GDM patients with BMI ≥ 25 had smaller cIMT that those with BMI < 25. This study regarded both BMI and metabolic syndrome as grouping criteria and BMI < 25 group also had no components of the metabolic syndrome. Metabolic syndrome may abolish this connection in this study.

We find that the diagnostic criteria of GDM may influence the impact of GDM on cIMT. Diagnosis of gestational diabetes significantly changed on the basis of the diagnostic criteria used, and influenced clinical outcomes [30, 31]. However, too few studies included in NDDG, WHO, ADA 75 g subgroups. In fact the NDDG criteria indicate more severe GDM than Carpenter-Coustan one. But we got no statistically different result in NDDG subgroup analysis, while a statistically different one in Carpenter-Coustan subgroup. The heterogeneity among different studies is relatively large, which may also cause this phenomenon.

The American Heart Association recommend to prevent heart disease in women with gestational diabetes, which was based on a higher risk of type 2 diabetes mellitus in these persons [32]. It is reported that cIMT adds predictive value to the Framingham risk score for cardiovascular events [5], is a level IIa recommendation for cardiovascular risk evaluation [33], cIMT has been confirmed to be able to predict incident coronary heart diseases [34]. Our finding that GDM is associated with early atherosclerosis even during pregnancy is important, because we can establish prevention strategy, such as weight control for GDM patients earlier in life.

Our research also finds increase of cIMT already exists at the time pregnancy. Another question raises our interests is that whether cIMT can predict GDM, as it’s demonstrated that cIMT is elevated before the onset of clinical diabetes [35]. However, cIMT measured prior to the pregnancy fails to predict pregnancy outcome such as gestational diabetes [36]. Thus subclinical atherosclerosis may appear along with GDM, but is not a predictor of GDM. We find cIMT does not increase years after GDM has been diagnosed. A possible explanation is that these patients take certain drugs to delay the process of atherosclerotic formation, it’s been reported that even subclinical atherosclerosis may be reduced by drugs [37]. As the medications of these patients were not fully reported in included studies, future researches are needed to study this issue.

The number of population in each study is limited; there was no study with number of GDM patients beyond 200. Prospective study of large samples is needed in the future.

## Conclusion

In this meta-analysis we observed GDM is related to larger cIMT, the relation is stronger in obese GDM patients, and the association already exists at the time of pregnancy and remained significant years after pregnancy. Weight control may be helpful to prevent cardiovascular diseases for GDM patients.

## Electronic supplementary material

Additional file 1: **Search strategy.** (DOCX 15 KB)

Additional file 2: Table S1: Supplemental characteristics of included studies. (DOC 50 KB)

## References

[1] Akinci B, Celtik A, Genc S, Yener S, Demir T, Secil M, Kebapcilar L, Yesil S (2011). Evaluation of postpartum carbohydrate intolerance and cardiovascular risk factors in women with gestational diabetes. Gynecol Endocrinol.

[2] Harreiter J, Dovjak G, Kautzky-Willer A (2014). Gestational diabetes mellitus and cardiovascular risk after pregnancy. Womens Health.

[3] Bauer M, Caviezel S, Teynor A, Erbel R, Mahabadi AA, Schmidt-Trucksass A (2012). Carotid intima-media thickness as a biomarker of subclinical atherosclerosis. Swiss Med Wkly.

[4] Touboul PJ, Vicaut E, Labreuche J, Acevedo M, Torres V, Ramirez-Martinez J, Vinueza R, Silva H, Champagne B, Hernandez-Hernandez R, Wilson E, Schargrodsky H, CS Investigators (2011). Common carotid artery intima-media thickness: the Cardiovascular Risk Factor Multiple Evaluation in Latin America (CARMELA) study results. Cerebrovasc Dis.

[5] Polak JF, Pencina MJ, Pencina KM, O’Donnell CJ, Wolf PA, D’Agostino RB (2011). Carotid-wall intima-media thickness and cardiovascular events. N Engl J Med.

[6] Zhang Y, Guallar E, Qiao Y, Wasserman BA (2014). Is Carotid Intima-Media Thickness as Predictive as Other Noninvasive Techniques for the Detection of Coronary Artery Disease?. Arterioscler Thromb Vasc Biol.

[7] Rademaker AA, Danad I, Groothuis JG, Heymans MW, Marcu CB, Knaapen P, Appelman YE (2013). Comparison of different cardiac risk scores for coronary artery disease in symptomatic women: do female-specific risk factors matter?. Eur J Prev Cardiol.

[8] Akinci B, Celtik A, Yener S, Genc S, Tunali S, Yuksel F, Ozcan MA, Secil M, Yesil S (2011). Plasma thrombin-activatable fibrinolysis inhibitor levels are not associated with glucose intolerance and subclinical atherosclerosis in women with previous gestational diabetes. Clin Appl Thromb Hemost.

[9] Akinci B, Celtik A, Yuksel F, Genc S, Yener S, Secil M, Ozcan MA, Yesil S (2011). Increased osteoprotegerin levels in women with previous gestational diabetes developing metabolic syndrome. Diabetes Res Clin Pract.

[10] Akinci B, Demir T, Celtik A, Baris M, Yener S, Ozcan MA, Yuksel F, Secil M, Yesil S (2008). Serum osteoprotegerin is associated with carotid intima media thickness in women with previous gestational diabetes. Diabetes Res Clin Pract.

[11] Akinci B, Celtik A, Tunali S, Genc S, Yuksel F, Secil M, Ozcan MA, Bayraktar F (2014). Circulating apelin levels are associated with cardiometabolic risk factors in women with previous gestational diabetes. Arch Gynecol Obstet.

[12] Vural M, Camuzcuoglu H, Toy H, Cece H, Aydin H, Eren MA, Kocyigit A, Aksoy N (2012). Evaluation of the future atherosclerotic heart disease with oxidative stress and carotid artery intima media thickness in gestational diabetes mellitus. Endocr Res.

[13] Eren MA, Vural M, Cece H, Camuzcuoglu H, Yildiz S, Toy H, Aksoy N (2012). Association of serum amyloid A with subclinical atherosclerosis in women with gestational diabetes. Gynecol Endocrinol.

[14] Atay AE, Simsek H, Demir B, Sakar MN, Kaya M, Pasa S, Demir S, Sit D (2014). Noninvasive assessment of subclinical atherosclerosis in normotensive gravidae with gestational diabetes. Herz.

[15] Bo S, Valpreda S, Menato G, Bardelli C, Botto C, Gambino R, Rabbia C, Durazzo M, Cassader M, Massobrio M, Pagano G (2007). Should we consider gestational diabetes a vascular risk factor?. Atherosclerosis.

[16] Caliskan M, Caklili OT, Caliskan Z, Duran C, Ciftci FC, Avci E, Gullu H, Kulaksizoglu M, Koca H, Muderrisoglu H (2014). Does Gestational Diabetes History Increase Epicardial Fat and Carotid Intima Media Thickness?. Echocardiography.

[17] Fakhrzadeh H, Alatab S, Sharifi F, Mirarefein M, Badamchizadeh Z, Ghaderpanahi M, Hashemi Taheri AP, Larijani B (2012). Carotid intima media thickness, brachial flow mediated dilation and previous history of gestational diabetes mellitus. J Obstet Gynaecol Res.

[18] Freire CM, Barbosa FB, De Almeida MC, Miranda PA, Barbosa MM, Nogueira AI, Guimaraes MM, Nunes Mdo C, Ribeiro-Oliveira A (2012). Previous gestational diabetes is independently associated with increased carotid intima-media thickness, similarly to metabolic syndrome - a case control study. Cardiovasc Diabetol.

[19] Gunderson EP, Chiang V, Pletcher MJ, Jacobs DR, Quesenberry CP, Sidney S, Lewis CE (2014). History of Gestational Diabetes Mellitus and Future Risk of Atherosclerosis in Mid-life: The Coronary Artery Risk Development in Young Adults Study. J Am Heart Assoc.

[20] Ijas H, Morin-Papunen L, Keranen AK, Bloigu R, Ruokonen A, Puukka K, Ebeling T, Raudaskoski T, Vaarasmaki M (2013). Pre-pregnancy overweight overtakes gestational diabetes as a risk factor for subsequent metabolic syndrome. Eur J Endocrinol.

[21] Ku YH, Choi SH, Lim S, Cho YM, Park YJ, Park KS, Kim SY, Jang HC (2011). Carotid intimal-medial thickness is not increased in women with previous gestational diabetes mellitus. Diab Metab J.

[22] Ozuguz U, Isik S, Berker D, Arduc A, Tutuncu Y, Akbaba G, Gokay F, Guler S (2011). Gestational diabetes and subclinical inflammation: evaluation of first year postpartum outcomes. Diabetes Res Clin Pract.

[23] Tarim E, Yigit F, Kilicdag E, Bagis T, Demircan S, Simsek E, Haydardedeoglu B, Yanik F (2006). Early onset of subclinical atherosclerosis in women with gestational diabetes mellitus. Ultrasound Obstet Gynecol.

[24] Vastagh I, Horvath T, Garamvolgyi Z, Rosta K, Folyovich A, Rigo J, Kollai M, Bereczki D, Somogyi A (2011). Preserved structural and functional characteristics of common carotid artery in properly treated normoglycemic women with gestational diabetes mellitus. Acta Physiol Hung.

[25] Volpe L, Cuccuru I, Lencioni C, Napoli V, Ghio A, Fotino C, Bertolotto A, Penno G, Benzi L, Del Prato S, Di Cianni G (2008). Early subclinical atherosclerosis in women with previous gestational diabetes mellitus. Diabetes Care.

[26] Yousefzadeh G, Hojat H, Enhesari A, Shokoohi M, Eftekhari N, Sheikhvatan M (2012). Increased carotid artery intima-media thickness in pregnant women with gestational diabetes mellitus. J Tehran Heart Cent.

[27] Sullivan SD, Umans JG, Ratner R (2012). Gestational diabetes: Implications for cardiovascular health. Curr Diab Rep.

[28] Sugiyama T, Nagao K, Metoki H, Nishigori H, Saito M, Tokunaga H, Nagase S, Sugawara J, Watanabe Y, Yaegashi N, Sagawa N, Sanaka M, Akazawa S, Anazawa S, Waguri M, Sameshima H, Hiramatsu Y, Toyoda N (2014). Pregnancy outcomes of gestational diabetes mellitus according to pre-gestational BMI in a retrospective multi-institutional study in Japan. Endocr J.

[29] Fadl H, Magnuson A, Ostlund I, Montgomery S, Hanson U, Schwarcz E (2014). Gestational diabetes mellitus and later cardiovascular disease: a Swedish population based case–control study. BJOG.

[30] Berggren EK, Boggess KA, Stuebe AM, Jonsson Funk M (2011). National Diabetes Data Group vs Carpenter-Coustan criteria to diagnose gestational diabetes. Am J Obstet Gynecol.

[31] Simmons D, McElduff A, McIntyre HD, Elrishi M (2010). Gestational diabetes mellitus: NICE for the U.S.? A comparison of the American Diabetes Association and the American College of Obstetricians and Gynecologists guidelines with the U.K. National Institute for Health and Clinical Excellence guidelines. Diabetes Care.

[32] Mosca L, Benjamin EJ, Berra K, Bezanson JL, Dolor RJ, Lloyd-Jones DM, Newby LK, Pina IL, Roger VL, Shaw LJ, Zhao D, Beckie TM, Bushnell C, D'Armiento J, Kris-Etherton PM, Fang J, Ganiats TG, Gomes AS, Gracia CR, Haan CK, Jackson EA, Judelson DR, Kelepouris E, Lavie CJ, Moore A, Nussmeier NA, Ofili E, Oparil S, Ouyang P, Pinn VW, Sherif K, Smith SC, Sopko G, Chandra-Strobos N, Urbina EM, Vaccarino V, Wenger NK (2011). Effectiveness-based guidelines for the prevention of cardiovascular disease in women–2011 update: a guideline from the American Heart Association. J Am Coll Cardiol.

[33] Greenland P, Alpert JS, Beller GA, Benjamin EJ, Budoff MJ, Fayad ZA, Foster E, Hlatky MA, Hodgson JM, Kushner FG, Lauer MS, Shaw LJ, Smith SC, Taylor AJ, Weintraub WS, Wenger NK, Jacobs AK, G American College of Cardiology Foundation/American Heart Asscoiation Task Force on Practice (2010). 2010 ACCF/AHA guideline for assessment of cardiovascular risk in asymptomatic adults: executive summary: a report of the American College of Cardiology Foundation/American Heart Association Task Force on Practice Guidelines. Circulation.

[34] Chambless LE, Heiss G, Folsom AR, Rosamond W, Szklo M, Sharrett AR, Clegg LX (1997). Association of coronary heart disease incidence with carotid arterial wall thickness and major risk factors: the Atherosclerosis Risk in Communities (ARIC) Study, 1987–1993. Am J Epidemiol.

[35] Hunt KJ, Williams K, Rivera D, O’Leary DH, Haffner SM, Stern MP, Gonzalez Villalpando C (2003). Elevated carotid artery intima-media thickness levels in individuals who subsequently develop type 2 diabetes. Arterioscler Thromb Vasc Biol.

[36] Harville EW, Viikari JSA, Raitakari OT, Juonala M (2012). Pregnancy complications and ultrasound measures of cardiovascular risk. Am J Epidemiol.

[37] D’Ascenzo F, Agostoni P, Abbate A, Castagno D, Lipinski MJ, Vetrovec GW, Frati G, Presutti DG, Quadri G, Moretti C, Gaita F, Zoccai GB (2013). Atherosclerotic coronary plaque regression and the risk of adverse cardiovascular events: a meta-regression of randomized clinical trials. Atherosclerosis.

[CR38] The pre-publication history for this paper can be accessed here:http://www.biomedcentral.com/1471-2261/14/132/prepub

