# Supplementary material for: Association of Gestational Diabetes Mellitus (GDM) with subclinical atherosclerosis: a systemic review and meta-analysis
Source: BMC Cardiovasc Disord. 2014 Sep 29;14:132. doi: 10.1186/1471-2261-14-132 (PMC4192280; doi:10.1186/1471-2261-14-132)
Supplement: Supplementary file 1 — Additional file 1: Search strategy. (DOCX 15 KB) [file 12872_2014_778_MOESM1_ESM.docx]

#1 "Diabetes, Gestational"[Mesh]

#2 "pregnancy induced diabetes" OR "gestational diabetes"

#3 "Carotid Intima-Media Thickness"[Mesh]

#4 "carotid intima-media thickness" OR "arterial wall thickness" OR "subclinical atherosclerosis"

#5 #1 OR #2

#6 #3 OR #4

#7 #5 AND #6

20 returned

#1 'pregnancy diabetes mellitus'/exp

#2 'arterial wall thickness'/exp

#3 #1 AND #2

47 returned
