# Supplementary material for: Association of Gestational Diabetes Mellitus (GDM) with subclinical atherosclerosis: a systemic review and meta-analysis
Source: BMC Cardiovasc Disord. 2014 Sep 29;14:132. doi: 10.1186/1471-2261-14-132 (PMC4192280; doi:10.1186/1471-2261-14-132)
Supplement: Supplementary file 2 — Additional file 2: Table S1: Supplemental characteristics of included studies. (DOC 50 KB) [file 12872_2014_778_MOESM2_ESM.doc]

Supp. Table 1. Supplemental characteristics of included studies.

| **athoru** | **Total cholesterol** | | **Triglyceride** | | **LDL-C** | | **HDL-C** | | **Parity** | |
| --- | --- | --- | --- | --- | --- | --- | --- | --- | --- | --- |
|  | **GDM** | **CG** | **GDM** | **CG** | **GDM** | **CG** | **GDM** | **CG** | **GDM** | **CG** |
| Baris Akinci 2014 | 196.06±37.90 | 177.49±31.71 | 119±66 | 89±57 | 120±33 | 102±27 | 52±13 | 58±15 |  |  |
| A.E. Atay 2013 | 221±32 | 158±25 | 205±35 | 150±59 | 142±28 | 78±24 | 43±5 | 49±5 | 3.14±1.5 | 2.1±1.6 |
| S. Bo 2007 |  |  | Group 1:  55 (36)  Group 2:  76 (47) | 69(47) | Group 1:  104±27  Group 2:  116±31 | 69(47) | Group 1:  62±8  Group 2:  50±12 | 69(47) | Group 1:  1.9±1.2  Group 2:  1.6±0.8 | 1.6±0.9 |
| Mustafa Caliskan 2014 | 188.7±27.1 | 181.8±32.1 | 130.7±44.5 | 125.4±58.4 | 118.1±23.9 | 111.0±30.3 | 45.9±9.4 | 45.7±10.6 |  |  |
| Mehmet Ali Eren 2012 | 210.36±49.88 | 201.47±19.72 | 234±92 | 220±82 | 114±41 | 104±22 | 50±10 | 54± 14 | 3.6 ± 2.5 | 2.5 ± 2.2 |
| Hossein Fakhrzadeh 2012 | 178.10±32.02 | 171.25±29.36 | 109.30±58.46 | 105.95±41.32 | 91.70±21.44 | 88.25±17.37 | 51.90±12.06 | 50.65±11.49 | 1.95±1.05 | 1.45±0.76 |
| Claudia Maria Vilas Freire 2012 | 189.72±5.09 | 171.86±3.74 | 142.05±15.23 | 75.58±3.74 | 114.21± 4.50 | 94.98±3.63 | 49.71 ± 1.83 | 59.19 ± 1.72 |  |  |
| Erica P. Gunderson 2014 | 174.9 (30.4) | 177.7 (31.4) | 68.8 (32.4) | 64.6 (37.1) | 106.2 (28.9) | 108.3(29.0) | 54.9 (13.0) | 54.9 (13.0) | 0.42 (0.67) | 0.42 (0.81) |
| H Ijas 2013 | 201±38.67 | 213±30.94 | 124±62 | 115±62 | 108±50 | 124±39 | 62±15 | 66±15 |  |  |
| Ufuk Ozuguz 2011 | 251.3±47.5 | 233.5±41.7 | 236.9±95 | 189.8±83.5 | 142.2±41.7 | 135.2±35.8 | 63.9±14. | 63.1±16.4 | 2.63±1.36 | 2.64±1.13 |
| E. TARIM 2006 | 242.2±33.46 | 229.6±41.57 | 225.55±66.03 | 190.38±48.32 | 129.93±31.27 | 127.86±46.31 | 65.76±17.95 | 63.91±24.18 |  |  |
| I Vastagh 2011 | 198.8±36.35 | 158.16±27.07 | 184±86 | 142±53 | 97±34 | 97±19 | 59±13 | 65±7 |  |  |
| Gholamreza Yousefzadeh 2012 | 219.0±54.7 | 223.8±43.8 | 183.5±67.0 | 178.0±61.0 | 140.7±37.4 | 137.7±36.0 | 49.0±8.7 | 49.0±10.5 |  |  |
| Volpe, L. 2008 |  |  | 91.28±67 | 62.7±21 |  |  |  |  |  |  |
| Yun Hyi Ku 2011 | 184.6±28.0 | 189.8±35.1 | 107.0(79.5-137.5) | 73.5(62.0-102.0) | 107.4±28.0 | 117.9±28.9 | 49.5 (43.3-60.0) | 49.0 (46.0-65.3) |  |  |
